# The Empirical Investigation Between Ethical Leadership and Knowledge-Hiding Behavior in Financial Service Sector: A Moderated-Mediated Model

**DOI:** 10.3389/fpsyg.2021.798631

**Published:** 2021-12-16

**Authors:** Muhammad Mohsin, Qiang Zhu, Xiaojun Wang, Sobia Naseem, Muhammad Nazam

**Affiliations:** ^1^School of Business, Hunan University of Humanities, Science and Technology (HUHST), Loudi, China; ^2^School of Economics and Management, Shijiazhuang Tiedao University, Shijiazhuang, China; ^3^Institute of Business Management Sciences, University of Agriculture, Faisalabad, Pakistan

**Keywords:** ethical leadership, knowledge hiding behavior, services sector, meaningful work, ethical climate

## Abstract

This study aimed to investigate the impact of ethical leadership on knowledge-hiding behavior of the employees working in the financial services sector under the mediating role of meaningful at work and moderating role of ethical climate. For this purpose, data were collected from two hundred and fifteen employees of financial services providing organizations. The already-established scales were followed to develop an instrument that was used to obtain responses from the respondents. Collected data were analyzed by applying the structural equation modeling through Smart PLS and Process Macro. The results indicate that ethical leadership and meaningful work (MW) reduce knowledge-hiding behavior of employees at work, while ethical leadership positively impacts the influential work of employees at the workplace. Further, the relationship between ethical leadership and knowledge-hiding behavior is partially mediated by MW. Similarly, ethical climate moderated the relationship between ethical leadership and knowledge-hiding behavior. This research makes valuable contributions to the existing literature on leadership and knowledge management. From a practical point of view, this study stresses that managers at work should promote ethical leadership styles to promote MW, which will reduce knowledge hiding. Thus, in this way, it will enhance the innovation and creativity within organizational circuits. The limitations and future directions of this study are also listed.

## Introduction

In a service organization, the impairment of interpersonal interactions and knowledge transfer processes may be more significant than the lack of expertise. There is also the continuity of execution and the fact that the resources must be provided and consumed in a close temporal partnership, resulting in information being sent and received concurrently (Awan et al., 2021). It is also that a dynamic and unpredictable demand structure involves great ingenuity and innovations to match the fast-to-changing consumer needs and informal sharing of information to satisfy these requests. Despite the adverse impact on service sector industries, the practice of information hiding is ignored chiefly. Companies have traditionally undervalued employees who possess advanced knowledge of their industry, industry-specific skills, and techniques instead of other corporate-level know-how and techniques. Few management interventions have been shown to challenge knowledge-hiding practices in the service sector. However, they encourage service workers to pursue. This has increased demands for more research on the phenomena of knowledge hiding in service firms (Fawehinmi et al., 2020).

The literature demonstrates that ethical leadership lessens the information hiding at the workplace (Banks et al., 2020). Thus, the role of workers in hiding knowledge may be disrupted by modeling their moral behaviors. However, there is little or no current research on the specifics of the inverse relationship between ethical leadership and knowledge hiding, mainly when covering up the mediators (Ouakouak et al., 2019). As a result, they leave a severe gap between our understanding about and where ethical leaders discourage their workers from participating in practices that function as a disservice to their colleagues (Saha et al., 2020). This research explores the missing investigations of previous research work on ethical leadership's responsible elements and information accessibility with knowledge hiding behavior (Abdillah et al., 2020). To expand upon the previously uncovered information, this work aims to bridge the gap. The present study identifies the intervening and co-constituting circumstances of ethical leadership and knowledge hiding in service sector firms (Irum et al., 2020).

In the view of previous theories on ethics, we suggest some fundamental mechanisms, i.e., meaningful work (MW) putting forward the effort to meet an impact; it is impactful, and actions in providing services can require a manager to hoard additional resources (Frémeaux and Pavageau, 2020). An association between information expansion, relational protection and ethical leadership on relational safety has emerged from the previous studies (Autin and Allan, 2020; Nikolova and Cnossen, 2020). This research shows a relationship between social capital and knowledge hiding. Previous studies have examined MW's impact on this interaction. But the nature of MW as a mediator of this connection has not been explored until now. Although studying the influence of MW is necessary for several reasons, we contend that conducting an investigation is needed (Lips-Wiersma et al., 2020). The existing literature says that focusing on increasing competitiveness has delivered good quality care and improved society's well-being. The aim is to force managers to look at financial benefits as purposeless and devoid of sense because it has turned them to only trying to be competent and wealthy rather than doing what's best for their clients (Both-Nwabuwe et al., 2020). As employees see their jobs as pointless, detached from reality, and view their work as superficial and insignificant, they become chronically cynical and lose interest in its context (You et al., 2020). Because of this, meaninglessness is now more often used as a severe problem in workplaces than ever before; this translates into an increasing need for organizations to research causes that assist with overcoming it (Brown et al., 2005; Wang and Lin, 2018; Albrecht et al., 2021).

Past studies have indicated that MW can be categorized as employees' fundamental right. Thus, it is imperative to explore the role of ethical leadership in promoting MW as a fundamental building block of ethical leadership style (Brown and Treviño, 2006). Thus, in previous studies, how ethical experience has contributed to skepticism has been minimally addressed, and the MW's importance as a motivator of these types of moral values has been overlooked (Singh et al., 2020). While this study uncovers how the current ethical leadership hindrance discourages workers from trying to keep what they've asked some knowledge, it prevents concerted efforts by employees to do. Efforts made in the manner seen above signal the importance of maintaining control of the organization's information, enabling future service managers to deal with threats to knowledge and provide a fresh outlook on guarding the organization's ability. The study's result helps to examine the relationship between ethical leadership and the fact that workers are not making themselves publicly available enough. It also has significant ramifications in the field of organizational knowledge-hiding environment. Additionally, the report resonates with the requirements to help companies solve the problems caused by competition in today's markets.

## Literature Review

### Knowledge Hiding

Knowledge hiding is a systematic information hoarding campaign to suppress, fabricate, and disseminate ambiguous, fabricated knowledge in the face of those who inquire about it (Butt and Ahmad, 2020). Unleaving the searchable information and providing believable answers is the rationale behind the expunging of mystery and ambiguity from inside hiders (Weng et al., 2020). We learned a good deal more about the subjects' details, but it was supplied in vague terms, as hiders might want to conceal or even fob us off the questioners. When a prospect feels as if they do not have the details requested, it is considered acting “dumb.” Many scholars use knowledge-hiding techniques that are conceptually diverse but still have a lot in common, such as obfuscation, redirection, misdirection, and isolation. Anand et al. (2020) discussed that hiding is always the case; regardless of matter how much the question asker tries, no one has been given what they asked for. It is likely to be a social dismissal and self-deception, though it may be intentional deception with many information seekers (Khoreva and Wechtler, 2020). Any undue restrictions on data flow of information or unclear instructions to the existence of intelligence can exist about the desired methods, or corresponding actions will have wide- and lasting-disastrous effects on both individuals and organizations (Jahanzeb et al., 2020).

Sometimes, a lack of awareness is to be used as an issue of deliberate exclusion. Sometimes, this results from a failure to contribute to the discussion; nevertheless, not having information doesn't include any malicious motive and therefore falls under the principle of ignorance (Ghani et al., 2020). Uncovered intentions to hide or distort information imply the possibility of intentional misrepresentation. It is often possible to hide information individually rather than on a societal ground; it is inspired by an antipathy to or interest in seeing how knowledge. It does not explicitly produce intent to educate; it does not learn independently; therefore, it is impulsive and inspired by a vague goal and hypothetical intentions to provide anonymous information (Abubakar et al., 2019). In some cases, individuals might tend to hide knowledge for personal enrichment to secure a unique position at the workplace.

### Theoretical Background

Stress is only one of the main stressors faced by individuals. Individuals obtain, maintain, safeguard, spend, and foster social capital, such as relations, conditions, and capabilities and their psychological and physical capabilities and their identities and abilities when accompanied by both loss and gain (Holmgreen et al., 2017). This extension of the conservation of resources theory states that workers' expected benefit and lack of resources impact job practices and significantly impact their working habits. Second, the available services in one area, such as work benefits, aid those necessary in other areas, such as well-being (Feng and Wang, 2019). Another aspect of the conservation resources theory; individuals want to maximize their wealth by re-allocating minimum resources. Also, those with more capital would express a higher degree of readiness to improve their reservoirs of resources. Specifically, they should put their money to use either though they believe they may get a net addition to their help or whether they are worried about the risk of losing net resources. The conservation of resources theory further suggests that leaders influence those who work under them in their organizations and that their leaders' feelings and actions may affect those who work under them (Halbesleben et al., 2014). From the theoretical lens of social identity theory, it can be safely concluded at the workplace that individuals with high knowledge might categorize themselves as different one from those who possess insufficient knowledge, thus increasing the likelihood of knowledge hiding because of their unique ability to help them constructs their self-concept and provide them with a professional identity. However, the ethical climate at the workplace has the potency to force individuals to stop knowledge hiding (Irum et al., 2020).

#### Linkages Between Ethical Leadership and Knowledge Hiding

Ethical leaders should show trustworthiness, justice, dependability, and regard for their followers' real needs and interests to be competent and valuable to others. Ethical leaders have services for their employees, but they also provide moral encouragement to their staff members so they can carry out their job (Men et al., 2018). Because of this, those employed under ethical control have access to services like emotional encouragement, personalized, and partnership support from their bosses. This means they would be less prone to emotional damage, as well as their productivity is raised. The conservation of resources theory notes that work capital, such as ethical leadership, may serve as a catalyst, leading to increases in personal ethics (Morelli and Cunningham, 2012; Li et al., 2021; Naiwen et al., 2021). It could very well be a contributor to a person's reservoir. The conservation of resources theory further argues that workers want to re-tread their dollars through the firm (Hobfoll and Freedy, 2017). They see either a capital opportunity or the possibility of no money being used up in the future. As workers' jobs are done under ethical officials, two things become apparent: They don't feel free to withhold information. They realize that the inquiry would be met with supervisor support rather than suspicion (Khalid et al., 2018). There are two main ideas in the argument that are as follows: A significant ethical leadership intervention focuses on all supporters and staff's interests by providing them with treatment and guidance in their personal and professional lives. However, a leader who supports their team would therefore be able to help while they are in need by offering them the resource they had previously. Second, giving other workers opportunities for what they are asked for may be considered expenditure in resources that can benefit them more resources and build stronger connections since it allows people to create something for them.

Conservation of resources theory, as well, states that constructive thoughts and actions influence others. Previous scientific research has proved that innovative behavior in the workplace will spread from one individual to another. Drawing from the principle of good attitudes, such as sincerity, sincere, and caring for others, we derive the ethical leadership's attributes: truthfulness, accountability, and compassion (Boz Semerci, 2019). Therefore, it can be argued that workers who express care for others and display concern for their colleagues are apt to satisfy their information demands since that they have specific needs already fulfilled. In other words, we often want workers to be frank with each other. For a long time, limited literature has proved that ethical leadership leads to decreased employee confidence in falsification and misdirection of facts. Additionally, ethical leaders must prevent their staff from concealing customer information (Zhu et al., 2019). Individuals who promote knowledge sharing at the workplace have prosocial intentions (Butt and Ahmad, 2020), while individuals who tend to hide knowledge have antisocial purposes (Irum et al., 2020). Thus, in the presence of an ethical leadership style, the probability to show antisocial behaviors at the workplace can be decreased, and individuals have to offer knowledge-sharing practices when the leader is highly ethical. Although leadership-based changes in attitudes and results may directly affect workers, we emphasize managers because of their impact on ethical standards and practices. The distinctness informs our commitment to supervisors' ethical leadership of their associations with their subordinates, the sheer amount of them, and their contact (Arshad and Ismail, 2018). Supervisors can work with the management to develop and promote management strategies and carry out their agendas. Bavik et al. (2018) claim that employers and managers should be aware of how they impact workers' processes and their progress by praise and punish their accomplishments.

**Figure 1:**
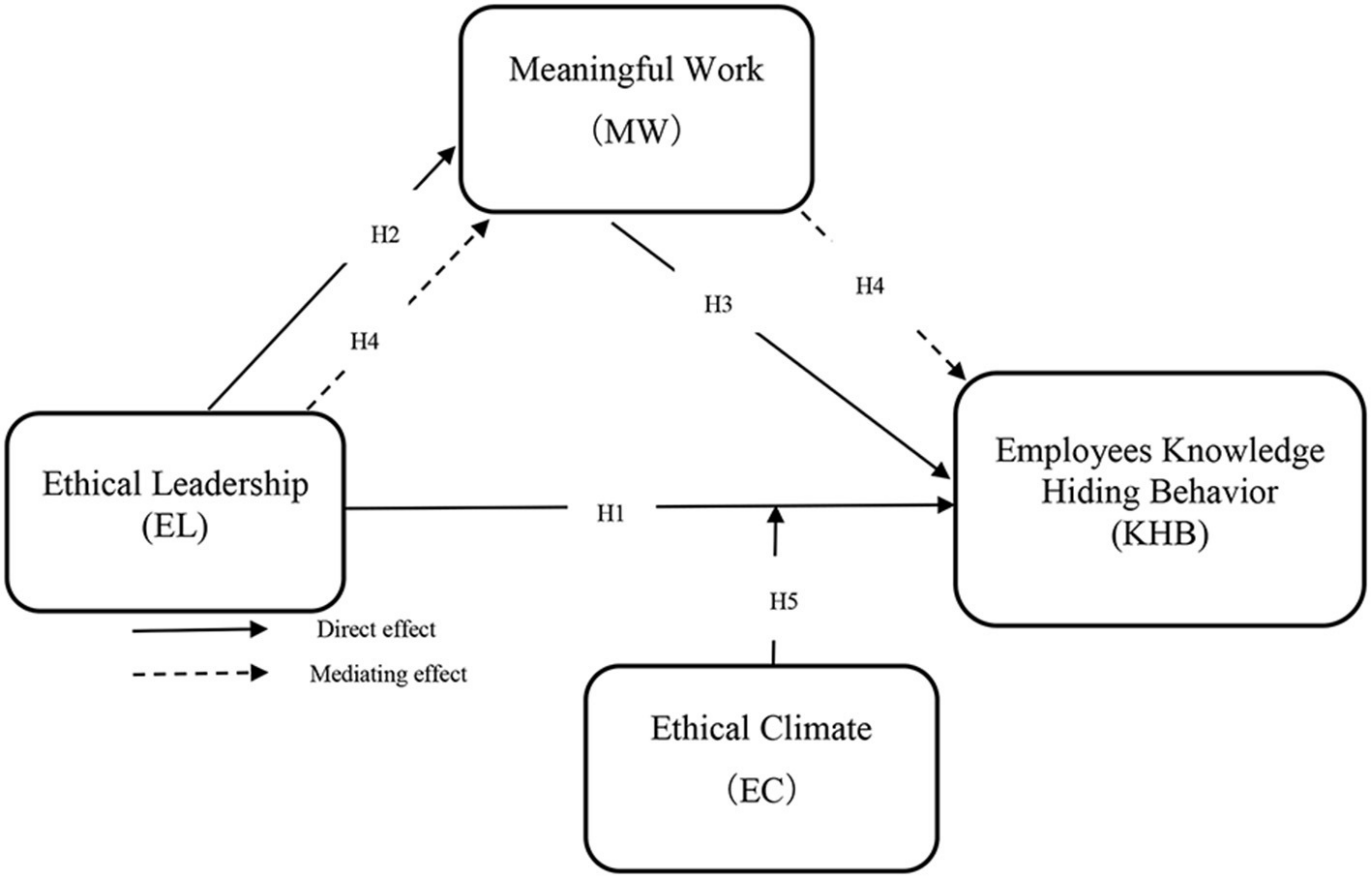
Conceptual framework.

Hence, the proposed hypothesis is as follows (see Figure 1):

*H*_1_: *Ethical leadership negatively affects employee's knowledge-hiding behavior*.

#### Ethical Leadership, Meaningful Work, and Knowledge Hiding

The MW identifies how workers see jobs and their skills as providing substantial benefits in their personal and professional lives and bringing other skills to bear. While the MW idea of separation has its historical origins in social estrangement notions, it is receiving renewed interest in organizational psychology, political science, market ethics, corporate social responsibility, and ideology (Demirtas et al., 2017). The scholarly practice thrives on results such as work participation and organization. Thus, the use of MW accounts has an essential effect on theories and practices. The reports overlook jobs' internal characteristics, as the work or mission occurs within a meaningful framework (Engelbrecht et al., 2014). Additionally, MW is a valuable personal resource.

Moreover, from the perspective of COR theory, MW can be termed as valuable personal resources. This resource can enrich the individuals to show positive behaviors at the workplace to promote helping behavior/culture. Therefore, based on COR, it can be safely assumed that ethical leadership being a resource will develop MW, which ultimately enables sharing knowledge and reducing knowledge hiding. Additionally, culture as a whole underestimates the function of position and organization. Thus, for those reasons presented in the above papers (Steger et al., 2012), see the idea of motivation-based well-being. As a multidimensional phenomenon that incorporates three distinctly interrelated yet connected factors, i.e., greater motivation, meaning-making, and well-being. For the concept of “true value” in work, the meaning is more significantly related to workers' beliefs about contributing to a greater good and productive cause (Abdullah et al., 2019). Therefore, a term for the notion that workers find meaning and development in their jobs is broadened, encompassing employees' thoughts about how they think they both see themselves and their environment surrounding them as they are doing it (Koay, 2018). The contributions to those that the worker feels their job makes and its importance for the system are also critical motivations for personal expansion. Hence, the proposed hypotheses are as follows (see Figure 1):

*H*_2_: *Ethical leadership positively affects meaningful work*.
*H*_3_: *Meaningful work negatively affects employee's knowledge-hiding behavior*.

#### The Mediating Role of Meaningful Work and The Moderating Role of Ethical Climate

What ethical leadership is a value-based strategy and envision is a more significant increased benefit for people. Culture and staff, as well as people's interests, are about accomplishing more significant objectives and executing socially aware operations and being a purpose-based entity to serve everyone. Business ethics is a vehicle for aligning a worker's sense of purpose with their workplace values, ensuring that their job performance and interests are congruent. Global view by work policies and procedures is developing new career designs and giving them the importance of specific jobs. The structural shaping of their perception of the work is included in this behavioral approach (Magnano et al., 2019). Furthermore, ethical leaders can affect their employees' sense of mission and value by equipping their workers with goals. Society and organizations ensure that their efforts, loyalty and help are meaningfully identifiable to their employees with specific roles to achieve the organization's goal (Zeglat and Janbeik, 2019). Ethical leadership improves workers' capacity to see various issues, for instance, “Who am I?” and “What is of importance to me?” to see questions such as “Who am I?” and “How does the background relate to my job?” and improving a sense of personal principles as features of “and who should I work with whom? matter to me?” From these premises, it can be deduced that ethical leadership can, in fact, positively impact the development of MW (Oprea et al., 2020).

Also, MW is a valuable source of personal information. Because conservation of resources theory assumes that acquiring resources in the context of desirable behavior traits enables valuable resources, rather than impeding the possibility of obtaining them, individuals may behave positively, and self-resources predict that future resources encourage future assets (Siahaan and Gatari, 2020; Li et al., 2021; Naiwen et al., 2021). Based on this, we believe that MW provides an ethical leadership that encourages others to provide the resources they request for potential resource growth. From other factors, one factor is the significant factor that is considered very important and impacts employee behaviors, which can be termed as organizational climate. This climate is based on the perception of organizational policies, practices, and procedures, both formal and informal. One component from this organizational climate is the ethical climate, reflecting three significant characteristics: egoism, utilitarianism, and deontology. Thus, employees working in ethical environments are considered moral when making decisions and setting examples of appropriate behavior. It can profoundly impact workplace behavior, attitudes, social interactions, and peer perceptions, which can promote knowledge sharing. Hence, the proposed hypotheses are as follows (see Figure 1):

*H*_4_: *Meaningful work mediates the negative relationship between ethical leadership and employee's knowledge-hiding behavior*.
*H*_5_: *Ethical climate moderates the relationship between the negative association of ethical leadership and employee's knowledge-hiding behavior under the mediating role of meaningful work*.

## Research Methodology

### Data Collection and Sample Size

The sampling frame of this study includes employees of the financial services sector of Pakistan. The financial service sector has long been a concern of both academia and practitioners. Scholars have realized that the financial services sector, in the shape of the banking sector, plays an essential role in developing the economy. Thus, it is imperative to explore the phenomena of acknowledging hiding in this sector. Keeping in view this concern, this study approached the employees of the banking sector for data collection. A well-structured questionnaire was distributed among employees using the non-probability convenience sampling technique—the listed banking sectors obtained from the Pakistan Stock Exchange. Before administrating the questionnaires to the respondents, formal approval was obtained from them, and they were assured that this study would only be used for academic purposes. The researcher personally visited the selected branches of banks and approached the respondents for data collection. The participation in the survey was on a volunteer basis. By keeping in view, the sample size of the study calculated by well-reputed and globally implemented sample size formula for focusing on a limited population Krejcie and Morgan (1970). According to the criteria recommended by Krejcie and Morgan (1970), a sample size of 384 is sufficient for an infinite population. To be on the safer side and improve the generalizability of the findings, questionnaires were delivered to 390 targeted respondents in different services firms, from which 240 questionnaires were received back, out of which 25 were partially filled which were discarded. Complete and usable questionnaires were considered with the final sample size of 215 respondents.

### Questionnaire and Measurements

A comprehensive literature review was conducted to ascertain the items observed regarding evaluating the relationship between latent variables. The questionnaire was developed and comprised of 19 questions in three parts by adopting items from different studies. On a Likert scale from one to five on all item scales, the respondents assessed their viewpoint. Ethical leadership was assessed with six items taken from the studies of Brown and Treviño (2006) on a 5-point Likert scale of one (strongly disagree) to five (strongly agree). Furthermore, MW was evaluated with six items taken from the study by Steger et al. (2012) on a 5-point Likert scale of one (strongly disagree) to five (strongly agree). On a 5-point Likert scale, knowledge-hiding behavior was measured with seven items adapted from the study by Connelly et al. (2012). Finally, the ethical climate was calculated based on four things developed by Cullen et al. (1993).

### Demographic Characteristics

The sample size of this analysis was 215 people. The participant was asked about their gender. The results show that most respondents were female, 93 in number, and 122 were male—questions on the age of respondents in the second question of the questionnaires. There are four divisions of the respondent age. The first section involves categories 21–30, 8% of the total participant. The second category is 31–40 years old, comprising 23.33% of the whole sample. The third category is 41–50 years, consisting of 36% of the survey respondents. The fourth section is over 51 or above, which is 71 consisting of 32.67%. The findings are shown in Table 1.

**Table 1:** Demographic characteristics of respondents.

**Measures**	**Items**	***N***	**% Age**
Gender	Male	122	56.67
	Female	93	43.33
Age	21–30	17	8.00
	31–40	50	23.33
	41–50	77	36.00
	51 and above	71	32.67
Education	Intermediate	36	16.67
	Bachelor	63	29.33
	Masters/M.Phil.	109	50.67
	PhD	7	3.33
Sector	Banking	54	25.00
	Insurance	32	15.00
	Security	32	15.00
	Teaching	43	20.00
	Lawyer	22	10.00
	Doctor	32	15.00
Marital status	Married	93	43.33
	Single	85	39.33
	Divorced	37	17.33
Income	0–35,000 PKR	29	13.33
	35,000–45,000	50	23.33
	45,000–55,000	73	34.00
	55,000–65,000	46	21.33
	65,000 and above	17	8.00

The analysis results show this frequency and percentage based on evaluations in tables and graphs. The results show that 36 respondents had an intermediate degree, 16.67% of the whole, 63 bachelors and 109 master/M—Phil, which is 80% of the total. The rest of 3.33% of respondents were PhD. The descriptive analysis further shows that 25% of the respondents were from the banking sector, 15% were from insurance, 15% were from securities companies, 20% were private-sector teachers, 10% were lawyers, and 15 % were doctors.

The marital status of respondents is divided into 3 classes: married, single, and divorced. The findings indicate that most of the respondents in this survey are single. Study findings indicate that 85 participants are single, with 43.33%; 93 respondents are married with a 39.33% response rate; and 37 respondents are divorced with 17.33%. The results are shown in Table 1. As the respondent monthly income are shown in Table 1, the monthly income of 29 respondents is 35,000, which accounts for 13.33% of the total respondent. Fifty respondents have income in between 35,000 and 45,000, accounting for 23.33% of total respondents. Respondents have income between 45,000 and 55,000 are 73 and showing 34% of total respondents. The findings show that 46 respondents have income between 55,000 and 65,000 which is 21.33% of the total. The number of respondents having income above 65,000 is 17, 8% of the total respondents.

## Results

### Measurement Model

**Figure 2:**
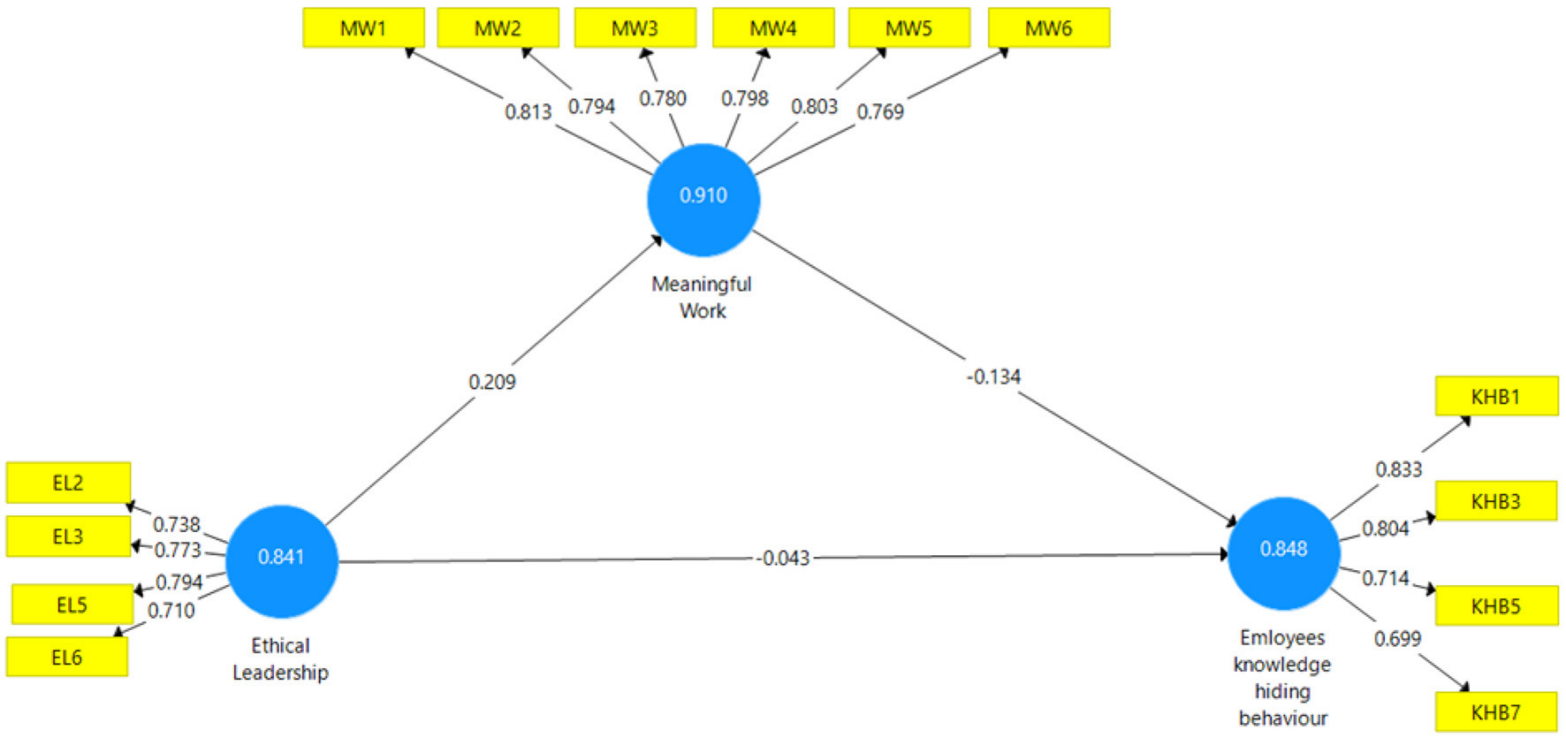
Construct reliability and validity.

A questionnaire survey methodology was used for data collection. This study examines the impact of “ethical leadership on employees' work-hiding behavior through the mediating role of MW.” The first step of analysis is to determine the model reliability and validity. Hence, for this purpose, Smart PLS was used for data analysis for several reasons; first, it deals very well with the small sample sizes. Second, it eradicates the issue of normality due to the sample bootstrapping technique (Khalid et al., 2018; Bashir et al., 2020; Mohsin et al., 2020; Naseem et al., 2021). For this purpose, construct reliability, discriminant validity, and convergent validity tests were used. According to our assessment of the reliability of the indicators, there are 14 indicators with outer loading >0.70 (Table 2, Figure 2). A total of four indicators of ethical leadership were used, all showing a reliable factor loading, as indicated in Table 2. The MW is measured through 6 items, which also show the factor loading >0.7. The knowledge-hiding behavior is measured through 4 items, and all the items' outer loading is more significant than 7, which is significantly reliable.

**Table 2:** Reliability and validity analysis.

**Variables**	**Constructs**	**Item**	**α**	**AVE**	***CR***
		**loading**			
Ethical leadership	EL2	0.738^***^	0.752	0.569	0.841
	EL3	0.773^***^			
	EL5	0.794^***^			
	EL6	0.710^***^			
Knowledge-Hiding behavior	KHB1	0.833^***^	0.766	0.584	0.848
	KHB3	0.804^***^			
	KHB5	0.714^***^			
	KHB7	0.713^***^			
Meaningful work	MW1	0.813^***^	0.883	0.629	0.910
	MW2	0.794^***^			
	MW3	0.780^***^			
	MW4	0.798^***^			
	MW5	0.803^***^			
	MW6	0.769^***^			

*CA ≥ 0.7; CR ≥ 0.7; AVE ≥ 0.5;*

*** *Significant threshold at p < 0.001*.

Structural equation modeling (SEM) has been used to explore the causal relationships and evaluate the conceptual framework (Henseler et al., 2009). In such a case, Smart PLS was used because (a) the early stage of theoretical evolution and (b) the uniqueness and complexity of the conceptual framework, the use of partial least squares (PLS) was deemed most suitable. First, we review the measurement model to determine the measurement reliability, consistency, convergent validity, and discriminative validity. We then evaluate the systemic connections in the conceptual model suggested. Our findings show that both Cronbach's alpha (CA) and composite reliability (CR) meet the appropriate standard of 0.7 and approve the reliability of constructs (Henseler et al., 2009). To determine the items' reliability, loadings should be >0.7 for all items (Henseler et al., 2009). All the factor loadings are more significant than 0.7, which confirms the reliability of the indicators. The findings also suggest that the average variance extracted (AVE) for constructs should be greater than above 0.5, confirming the convergent validity of constructs (Table 2). Finally, three parameters were used to evaluate the discriminant validity.

### Convergent and Discriminant Validity

Three criteria were used for the assessment of discriminant validity. The first criterion is the Fornell–Larcker criterion, which requires that the square root of AVE should be greater than the correlation to every other latent variable for each latent variable (Fornell and Larcker, 1981). These conditions are satisfied, as illustrated in Table 2. Second, each indicator's loading should be greater than all its cross-loadings. In the “heterotrait–monotrait ratio” (HTMT), all the values of constructs are less than the threshold level, which is 0.9 (Henseler et al., 2015; Li et al., 2021; Naiwen et al., 2021). Finally, the evaluation of the reliability of constructs, convergent and discriminant validity shows that all constructs are reliable and valid and used to examine the model.

The HTMT, as shown in Table 3, is also used to assess the discriminant validity of the model. All the values should be smaller than 0.9 (Henseler et al., 2015). The reliability assessment, convergent validity, and reliability of an indicator imply satisfactory results, which indicate that the theoretical model can be tested by constructs.

**Table 3:** Convergent validity.

**Construct**		**Convergent validity**					**Fornell larcker**		
	**Mean**	**SD**	**α**	**AVE**	**CR**	**1**	**2**	**3**	
1	Employees knowledge-hiding behavior	0.227	0.037	0.766	0.584	0.848	0.764		
2	Ethical leadership	0.757	0.014	0.752	0.569	0.841	−0.071	0.754	
3	Meaningful work	0.595	0.036	0.883	0.629	0.910	−0.143	0.209	0.793

This study measured the variance inflation factor (VIF) values to test Framework's collinearity problems. According to the experts, if the VIF value is <5, the findings are free of collinearity (Sarstedt et al., 2014). The inner VIF values for all constructs found that the objects range from 1.214 to 1.674. The current study's results show that there is no collinearity issue with the data and that the survey findings are stable (see Table 4).

**Table 4:** Discriminant validity.

	**Constructs**	**1**	**2**	**3**
1	Employee's knowledge hiding behavior	0.897		
2	Ethical leadership	−0.854	0.876	
3	Meaningful work	−0.785	0.786	0.876

### Predictive Accuracy

A bootstrapping of 5,000 samples was used to evaluate the hypothesis (Tenenhaus et al., 2005). The structural model was evaluated by the R-square and the Q-square, as Hair et al. (2016) proposed. The coefficient of determination (R-square) is considered moderate when the R-square value should be above 0.33. The R-square is 52.9% for MW and 72.3% for employees' knowledge-hiding behavior, suggesting that the frameworks have medium predictive accuracy. The positive values of Q-square were also endorsed. Hair et al. (2014) suggest that the “cross-validated redundancy” *Q*^2^ test was used to determine the predictive relevance of the model (see Table 5). The acceptable value of *Q*^2^ must be >“0.” Therefore, all the values of Q-square are more significant than 0. However, the value of *Q*^2^-values of two exogenous constructs indicates that we prove the high predictive accuracy of the research framework (Hair et al., 2016). Therefore, the researchers argue that *R*^2^ is used to evaluate the model predictive accuracy power (Henseler et al., 2015).

**Table 5:** Predictive accuracy and relevance of the model.

	**R Square**	**R square adjusted**
Meaningful work	0.529	0.525
Knowledge hiding behavior	0.723	0.742

**Figure 3:**
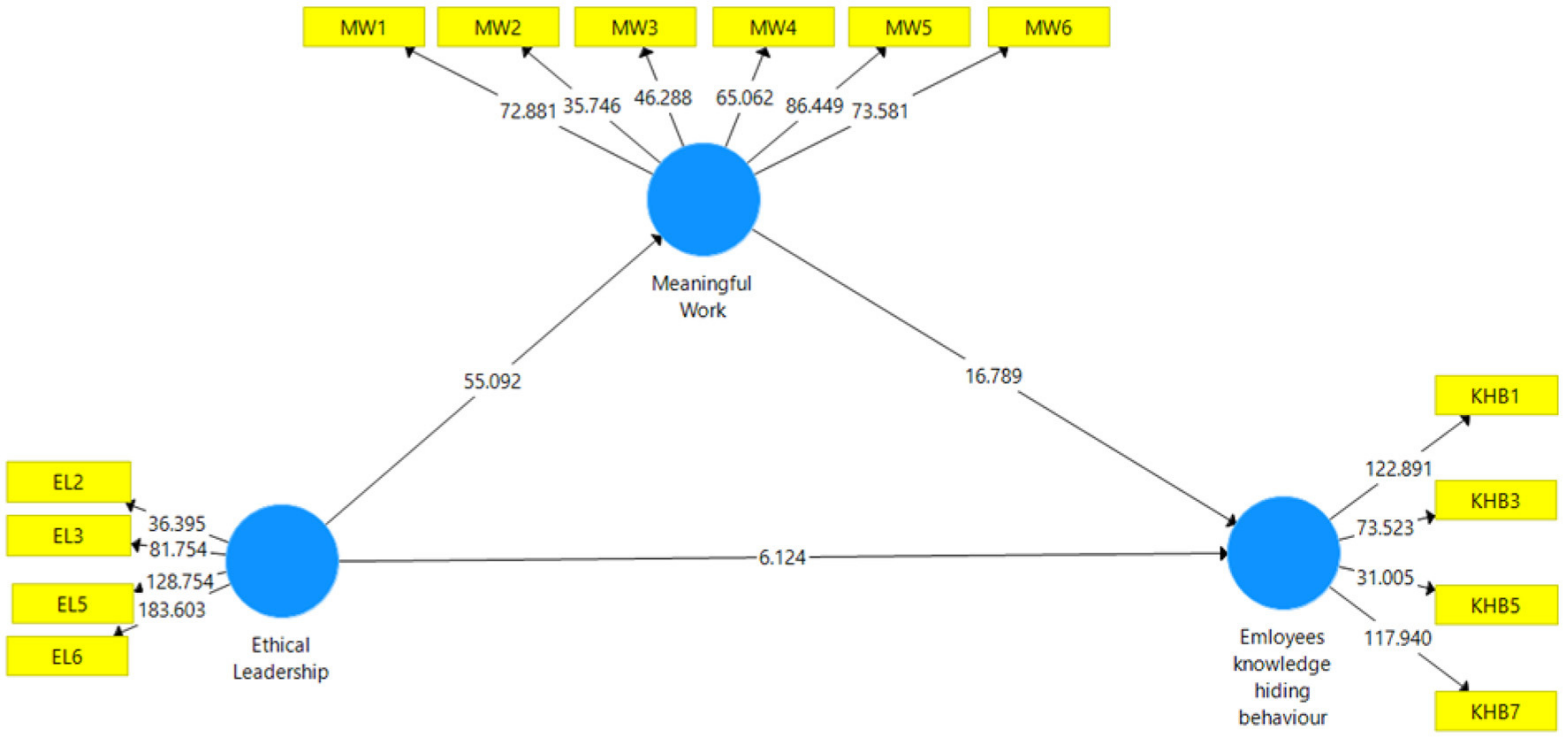
Structural model.

As purposed, ethical leadership negatively influence the employee's knowledge-hiding behavior. The result of this study shows the negative impact of ethical leadership on knowledge-hiding behavior (β = −0.043, *t*-value = 6.12, *p* = 0.000). These findings support Hypothesis 1. H2 hypotheses that ethical leadership has a positive impact on MW behavior. The result shows that ethical leadership has a significant and positive impact on MW behavior (β = 0.209, *t*-value = 55.09, *p* = 0.000). These results approved the H2 hypothesis. H3 purposed as the MW behavior has a negative relationship with employee's knowledge-hiding behavior. The result shows that MW has a significant negative impact on knowledge-hiding behavior (β = −0.134, *t*-value = 16.68, *p* = 0.000). So the H1, H2, and H3 hypotheses are approved (see Table 6, Figure 3). This research explores the missing investigations of previous research work on ethical leadership's responsible elements and information accessibility with knowledge hiding behavior (Abdillah et al., 2020).

**Table 6:** Hypothesis testing.

**Hypothesis**		**Path**	**ST**	***T***	**P-Value**	**Findings**
		**coefficient**	**statistics**			
H1	EL —> KHB	−0.043	0.037	6.12	0.000	H1, Supported
H2	EL —> MW	0.209	0.014	55.09	0.000	H2, Supported
H3	MW —> KHB	−0.134	0.036	16.78	0.000	H3, Supported

### Mediation Analysis

According to Meng and Bari (2019), the mediating role of MW is between ethical leadership and knowledge-hiding behavior through variance accounted for (VAF) technique. If the value of VIF is >80%, then it shows complete mediation. However, Meng and Bari (2019) suggest that the VIF value falls in between 20 and 80%, which indicates the partial mediation, and if the value of VIF falls below 20%, then there is no mediation.

Concerning increasing our knowledge about the relationship between “ethical leadership” and “knowledge-hiding behavior,” we determine the mediating role of MW, which is indirect impact. The entire mediation is accomplished when the direct relation is insignificant through the intervening of indirect influence. However, “partial mediation” is found when both direct and indirect effects are significant (Zhao et al., 2010; Gul et al., 2021; Jamil et al., 2021).

The results are shown in Table 7 that there is a partial mediation role of MW between “ethical leadership” and “knowledge-hiding behavior.” The findings show that the direct impact of “ethical leadership” on “knowledge-hiding behavior” is significant (β = −0.043, *t*-value = 6.12, *p* = 0.000). The direct impact remains significant with the intervention of MW as a mediator (β = 0.452, *t*-value = 15.26, *p* = 0.000). The managers can dissuade service personnel from engaging in knowledge-hiding activities and create a long-term competitive advantage by showing ethics and enhancing workers' senses of MW (Anser et al., 2021).

**Table 7:** Mediation effect.

**Relationship**	**Direct effect**	**Indirect effect**	**Total effect**	**VAF**	**Interpretation**	**Findings**
EL -> MW -> KHB	−0.043 (6.12)	0.452 (15.26)	0.677 (36.40)	67.12	Partial Mediation	H4, Supported

### Moderation Analysis

Table 8, in case of moderation analysis impact of interaction term (InT-1), has been found significant (LLCI = −0.3118 and ULCI = −0.0121), which indicates that the prevalence of ethical climate can reinforce positive behaviors and individuals at work can discourage knowledge hiding. Pagliaro et al. (2018) found that the ethical organizational climate of self-interest emphasizes an individualistic approach to ethical concerns. These ethical approaches decrease effective organizational affiliation and improve a cognitive process of moral disengagement.

**Table 8:** Moderation analysis.

**Predictors**	**Coeff**.	**se**	***t***	***p***	**LLCI**	**ULCI**	**Findings**
Constant	1.235	0.559	2.210	0.0282	0.1334	2.3362	H5, Supported
Int_1	−0.162	0.076	−2.131	0.0343	−0.3118	−0.0121	
	***R*** **^2^-chng**	***F***	**df_1_**	**df_2_**	***p***		
X*W	0.021	4.541	1.000	210.000	0.0343		

*Moderating variable = ethical climate*.

## Discussion and Conclusion

Our research found a clear inverse relationship between ethical leadership and knowledge-hiding behavior among our service staff members. This research indicates a lack of connection between these two ideas. According to our findings, leaders lead to more ethical progress (Irum et al., 2020). Working for an organization led by service-oriented leaders inculcates an ethic of openness, and the staff is less inclined to secrecy. Ethical leadership's traits, such as honesty and fairness, discourage followers from getting lost in the knowledge, which prevents knowledge from corrupting ethical engagement. These findings parallel the previous study of Boz Semerci (2019). The author also presented in his studies that if the leader has ethical norms, it positively impacts employee behavior within the organization, and employees start to contribute positive input by sharing their knowledge and experience with other coworkers. This positive behavior leads to the significant growth of the organization. Conservation of resource theory also confirms our findings.

According to our findings, MW mediates the inverse relationship between knowledge-hiding behavior by service employees and a sense of ethical responsibility. These findings indicate ethical leadership's behaviors and actions that embrace honesty, fairness, and a desire to help other stakeholders develop. Furthermore, its contribution to their personal and professional well-being develops service employees' feelings of MW, contributing to their organization and society (Oprea et al., 2020). Our findings are consistent with the previous study of Siahaan and Gatari (2020). They presented that meaningful is a positive element in the organization, which helps employees to focus on their work and share positive knowledge with their colleagues.

This study also presents the moderating effect of the organization's ethical climate on the relationship of ethical leadership and knowledge-hiding behavior of employees. If an organization focuses on developing an ethical climate, it helps build positive behavior among its employees. Our findings are parallel with the prior study. They concluded that if an organization provides an ethical climate to its employees, it creates a sense of positivity in the minds of its employees, and this positivity leads to a good relationship among all its employees. Furthermore, they start to help each other by sharing their knowledge and experience.

### Theoretical Contributions

As an additional contribution to several disciplines, this present work produces new knowledge. Previous research has demonstrated that social capital and psychological safety affect knowledge concealment (Albrecht et al., 2021). Furthermore, it is believed that MW serves as a link between “ethical leadership” and “knowledge hiding” (You et al., 2020). Therefore, ethical leadership had a noticeable impact on service workers' knowledge-hiding behavior, as noted in the current study.

We are pleased to be making our second contribution to the research on MW. The general literature on meaningfulness may improve the efficiency of the organization's job-effectiveness in several ways (Butt and Ahmad, 2020). Thus, they find a more successful work–family balance. However, to the best of our knowledge, the literature has not looked at MW and knowledge hiding concerning leadership and ethical leadership, but rather the corporate literature has compared it to analytical or innovative thinking (Feng and Wang, 2019; Ramzan et al., 2021). Our research enhanced the general study and service literature on the relationship between ethical leadership and knowledge-hoarding behavior among banking service employees. By conducting that research, the present work discovered a hidden mechanism through which an ethical leader influences service workers to keep them from using knowledge to shield their customers from future misfortune. While it may fit in with recent trends to enhance the nomological networks of the concept of MW, our research denied those (Khalid et al., 2018).

### Practical Implications

Our work allows us to understand better the relationship between banking service organizations and employees' knowledge-hiding behavior. The results indicate that supervisor intervention is vital in avoiding these antisocial behaviors. To meet this goal, supervisors act ethically and communicate their responsibility in such a way that it has a universal and spiritual application. When you're in charge, it's essential to have empathy and promote an understanding of others' personal and professional needs so you can contribute to the well-being of others, including proficient knowledge. Managers should hold additional pieces of training to strengthen the capabilities needed to encourage the further development of the organization's service development efforts, especially about the requested services and know-how. Keeping things secret or altering the facts does more harm to services than anything else. Supervisors should advise their workers that such behavior can hinder the quality and service delivery primarily because of the simultaneous production and consumption of services. In addition, supervisors should point out that this leads to productivity and relationship issues, which impedes smooth communication with concurrent services.

Managers must meet with their employees to better understand their value system and ask for their insights to aid in that process. The bottom line is that these casual conversations allow supervisors to zero in on a problem to cultivate a sense of motivation in their workers. These sessions can help managers put the importance of work on a higher social and corporate level and assist them in conveying that it is socially and institutionally significant. This way, supervisors can put a better and more lasting mark on the service workforce by making employees fully aware of the importance of their service contributions. Developing genuine reasons for their professional self-interest will inspire service-focused motivations and increase their devotion to the banking sector. Reducing engagement in knowledge-hiding behaviors in the workplace would help managers do their jobs better.

## Data Availability Statement

The original contributions presented in the study are included in the article/supplementary material, further inquiries can be directed to the corresponding authors.

## Author Contributions

All authors listed have made a substantial, direct, and intellectual contribution to the work and approved it for publication.

## Funding

This work was supported by the construct program of applied characteristic discipline Applied Economics Hunan Province.

## Conflict of Interest

The authors declare that the research was conducted in the absence of any commercial or financial relationships that could be construed as a potential conflict of interest.

## Publisher's Note

All claims expressed in this article are solely those of the authors and do not necessarily represent those of their affiliated organizations, or those of the publisher, the editors and the reviewers. Any product that may be evaluated in this article, or claim that may be made by its manufacturer, is not guaranteed or endorsed by the publisher.
